# Incidence and Predictors of Pregnancy among a Cohort of HIV-Positive Women Initiating Antiretroviral Therapy in Mbarara, Uganda

**DOI:** 10.1371/journal.pone.0063411

**Published:** 2013-05-21

**Authors:** Angela Kaida, Lynn T. Matthews, Steve Kanters, Jerome Kabakyenga, Conrad Muzoora, A. Rain Mocello, Jeffrey N. Martin, Peter Hunt, Jessica Haberer, Robert S. Hogg, David R. Bangsberg

**Affiliations:** 1 Simon Fraser University, Burnaby, Canada; 2 Beth Israel Deaconess Medical Center, Boston, Massachusetts, United States of America; 3 Harvard Medical School, Boston, Massachusetts, United States of America; 4 Massachusetts General Hospital, Boston, Massachusetts, United States of America; 5 University of British Columbia, Vancouver, Canada; 6 Mbarara University of Science and Technology, Mbarara, Uganda; 7 University of California San Francisco (UCSF), San Francisco, California, United States of America; 8 BC Centre for Excellence in HIV/AIDS, Vancouver, Canada; University of Washington, United States of America

## Abstract

**Objective:**

Many people living with HIV in sub-Saharan Africa desire biological children. Implementation of HIV prevention strategies that support the reproductive goals of people living with HIV while minimizing HIV transmission risk to sexual partners and future children requires a comprehensive understanding of pregnancy in this population. We analyzed prospective cohort data to determine pregnancy incidence and predictors among HIV-positive women initiating antiretroviral therapy (ART) in a setting with high HIV prevalence and fertility.

**Methods:**

Participants were enrolled in the Uganda AIDS Rural Treatment Outcomes (UARTO) cohort of HIV-positive individuals initiating ART in Mbarara. Bloodwork (including CD4 cells/mm^3^, HIV viral load) and questionnaires (including socio-demographics, health status, sexual behavior, partner dynamics, HIV history, and self-reported pregnancy) were completed at baseline and quarterly. Our analysis includes 351 HIV-positive women (18–49 years) who enrolled between 2005–2011. We measured pregnancy incidence by proximal and distal time relative to ART initiation and used multivariable Cox proportional hazards regression analysis (with repeated events) to identify baseline and time-dependent predictors of pregnancy post-ART initiation.

**Results:**

At baseline (pre-ART initiation), median age was 33 years [IQR: 27–37] and median prior livebirths was four [IQR: 2–6]. 38% were married with 61% reporting HIV-positive spouses. 73% of women had disclosed HIV status to a primary sexual partner. Median baseline CD4 was 137 cells/mm^3^ [IQR: 81–207]. At enrolment, 9.1% (31/342) reported current pregnancy. After ART initiation, 84 women experienced 105 pregnancies over 3.8 median years of follow-up, yielding a pregnancy incidence of 9.40 per 100 WYs. Three years post-ART initiation, cumulative probability of at least one pregnancy was 28% and independently associated with younger age (Adjusted Hazard Ratio (AHR): 0.89/year increase; 95%CI: 0.86–0.92) and HIV serostatus disclosure to primary sexual partner (AHR: 2.45; 95%CI: 1.29–4.63).

**Conclusions:**

Nearly one-third of women became pregnant within three years of initiating ART, highlighting the need for integrated services to prevent unintended pregnancies and reduce periconception-related risks for HIV-infected women choosing to conceive. Association with younger age and disclosure suggests a role for early and couples-based safer conception counselling.

## Introduction

In sub-Saharan Africa, the majority of new HIV infections occur in women of reproductive age [1]. Studies in North America [2], [3], Europe [4], [5], [6], and sub-Saharan Africa [7], [8], [9], [10], [11], [12], [13], [14], [15] consistently report that HIV-infected women and men desire children. HIV-uninfected individuals who seek to conceive with an HIV-infected partner risk acquiring HIV. If conception does occur, pregnancy itself is associated with increased risks of HIV acquisition and transmission [16], [17], [18].

HIV-infected women and men who desire biological children require strategies to protect at-risk partners and future children from infection [13], [19], [20]. Antiretroviral treatment for the infected partner [21], topical or systemic pre-exposure prophylaxis for the uninfected partner [22], [23], [24], [25], and sex without condoms limited to peak fertility offer effective safer conception strategies. In addition, sperm processing and insemination for male-infected couples [26], [27], and home insemination [28] and/or male circumcision [29], [30], [31] for female-infected couples reduce sexual transmission risk for HIV-serodiscordant couples [32], [33]. When HIV-positive individuals do not desire children, effective contraception is crucial to prevent unintended and/or unwanted pregnancies [34], [35], [36].

Successful translation of HIV-prevention strategies that acknowledge the reproductive goals and rights of people living with HIV [37], [38], [39], [40] requires a comprehensive understanding of pregnancy incidence and predictors among HIV-infected and at-risk women. These data are critical to estimate size, characteristics, and needs of priority target populations to support safer conception and contraception, maternal and child health, and HIV treatment and care programs [10], [41], [42].

Uganda is an important setting in which to investigate pregnancy incidence given high fertility rates (total fertility rate = 6.7 births per women [43]), endemic HIV (adult HIV prevalence = 6.7% [1]), and expanding antiretroviral therapy coverage (47% of people eligible for treatment [44]).

The primary objective of this prospective study was to estimate pregnancy incidence and assess baseline and time-updated predictors of pregnancy among reproductive-aged women enrolled in a cohort of HIV-positive individuals initiating ART in a rural region of Uganda.

## Methods

### Study Setting

Mbarara is a rural setting (population 83,700) located approximately 265 kilometres southwest of Kampala. Adult HIV prevalence in the region is estimated at 10% [45]. The Mbarara University HIV clinic, called the Immune Suppression Syndrome (ISS) clinic, is situated within the Mbarara Regional Referral Hospital. The clinic has served more than 18,000 patients since it opened in 1998 and offers comprehensive HIV care services, including ART, at no cost to patients. ART is provided through the Ugandan Ministry of Health with support from the President’s Emergency Plan for AIDS Relief (PEPFAR), the Global Fund, and the Family Treatment Fund [46].

### Study Participants

Study participants were enrolled in the Uganda AIDS Rural Treatment Outcomes (UARTO) cohort study, which was initiated in July 2005 with the primary objective of determining predictors of virologic failure and antiretroviral resistance. Participants were recruited from treatment-naïve patients initiating ART at the HIV clinic. Clinic patients who were at least 18 years old and living within 60 kilometers of the clinic were eligible to enroll in the study. At the time of this analysis, 500 individuals were enrolled in UARTO and 94% initiated ART within four days of enrolment. The loss-to-follow-up rate (participants for whom we were unable to confirm vital status after > = 180 days without cohort follow-up) among UARTO participants was 2% at one year and 5% at two years. For this analysis of pregnancy incidence, the sample was restricted to female UARTO participants aged 18–49 years.

Over the cohort follow-up period, national antiretroviral treatment guidelines were updated twice. Current (2009) guidelines recommend treatment for HIV-infected adults with CD4 cell count below 250 cells/mm^3^, or below 350 cells/mm^3^ for those with tuberculosis, pregnancy, or WHO stage III or IV disease [47]. Guidelines for participants who initiated treatment prior to 2009 recommended ART initiation at CD4<200 cells/mm^3^ or WHO Stage IV disease [48], [49].

### Data Collection

UARTO participants completed a baseline interview and phlebotomy. They were subsequently scheduled for quarterly follow-up interviews and phlebotomy, concurrent with their scheduled clinic visits. Standardized interviewer-administered questionnaires detailed demographics, mental and physical health, sexual risk behaviour, and partner dynamics including partner testing and HIV status. Incident pregnancies (and pregnancy outcomes) were assessed quarterly via female participants’ self-report. Interviews took 35–50 minutes to complete and were conducted by trained interviewers fluent in English and Runyankole, the dominant local language.

This analysis includes data from participants enrolled from June 2005 and followed-up through December 2011.

### Measures

The primary outcome was self-reported pregnancy at baseline and over the follow-up period (i.e., post ART-initiation), including both first and recurrent pregnancies. Incidence was computed using standard person-time methods. We applied the following rules to calculate woman-years ‘at risk’ for pregnancy: (1) Among women who reported pregnancy at baseline, time at risk commenced upon the first subsequent visit where they reported no longer being pregnant; (2) Women who became pregnant during follow-up were censored upon their first visit reporting the pregnancy and uncensored upon their first visit reporting no longer being pregnant; (3) Women who did not become pregnant were censored at the end of the follow-up period; and (4) Women who reported sterilization (tubal ligation or hysterectomy) were censored at baseline or, if during follow-up, upon reported date of the procedure.

We examined the association of incident pregnancy subsequent to ART initiation with baseline and time-updated variables, identified as covariates of pregnancy incidence in previous studies [8], [10], [42], [50], [51], [52], [53], [54], [55]. Baseline variables included socio-demographic characteristics (including age, education, employment, household income, and marital status), reproductive history (including parity), and clinical history (including time since HIV diagnosis, time on ART, AIDS defining illnesses, CD4 cell count at enrolment, and body mass index (BMI)). Time-updated variables were measured quarterly and included CD4 cell count, HIV viral load <400 copies/mL, depression (measured using a modified version of the Hopkins Symptom Check List and a cut-off of ≥1.75 as indicative of clinical depression) [56], [57], [58], the Medical Outcomes Study HIV Health Survey (MOS-HIV) Physical Health and Mental Health Summary scores (scored on a 0–100 scale, where a higher score indicates better health) [59], [60], sexual activity in the previous three months, and disclosure of HIV status to primary sexual partner. ‘Disclosure to primary sexual partner’ was included as a time-updated variable, which combined information on currently having a primary sexual partner (including spouse or regular partner) and disclosure of HIV status to that partner. This yielded a three-level variable including, (i) having disclosed HIV serostatus to a primary sexual partner; (ii) not having disclosed to a primary sexual partner; or (iii) not having a primary sexual partner (disclosure not applicable). In longitudinal analyses, HIV serostatus disclosure was time-updated based on changes in relationship status.

We also report pregnancy outcomes, based on participant self-report. The outcome categories include live birth or “stillbirth/miscarriage/termination” – the latter category includes three possible outcomes asked as one composite outcome measure in the interviews.

### Statistical Analyses

Baseline characteristics of women with and without pregnancies subsequent to ART initiation were compared using Wilcoxon rank sum test for continuous variables and Fisher’s exact test for categorical variables.

We computed baseline pregnancy prevalence (all pregnancies reported at or within 12 months prior to cohort enrolment), cumulative incidence of pregnancy, and pregnancy outcome post ART-initiation. Pregnancy incidence was calculated using person-time methods and is reported as number of pregnancies per 100 woman-years (WYs) of follow-up. Time between cohort enrolment to pregnancy or end of follow-up period was calculated for each participant. We present data on overall pregnancy incidence as well as pregnancy incidence (overall and first pregnancy) within specified time intervals to distinguish between proximate and distal associations between ART initiation and pregnancy incidence.

Kaplan-Meier curves display trends in pregnancy incidence over time stratified by key baseline characteristics, including age, marital status, and HIV serostatus disclosure to primary sexual partner. Log-rank and likelihood ratio tests were used to test differences in curves by predictor variable strata.

We modeled repeated events in a survival analysis with time-dependent covariates using Cox proportional hazards regression to identify independent predictors of pregnancy subsequent to ART initiation. The modified sandwich estimator was used to account for repeated measures among women with more than one pregnancy during follow-up [61]. Follow-up time began at treatment initiation or at first non-pregnant visit for women who were pregnant at treatment initiation (i.e., baseline). After testing for co-linearity and interactions, variables with significant association with pregnancy in the bivariate analysis were considered for the full model to obtain the relative contribution of each covariate, expressed as an adjusted hazard ratio (AHR) with a 95% confidence interval. Model selection was achieved by minimizing the Akaike information criterion (AIC) while maintaining p-values for covariates below 0.20 [62]. All statistical tests were 2-sided and were considered significant at α = 0.05. Data were analyzed with SAS version 9.3 [63].

### Ethical Statement

All participants provided voluntary, written informed consent at study enrolment. All procedures were approved by the Institutional Ethics Review Board of Mbarara University of Science and Technology (MUST), the Uganda National Council on Science and Technology (UNCST), Partners Human Research Committee, and the Research Ethics Board of Simon Fraser University.

## Results

### Baseline Characteristics

351 women aged 18–49 years with baseline data were eligible for this study. Analysis of incident pregnancy and predictors was restricted to 314 women with at least one follow-up visit, who contributed 1117.6 woman-years (WYs) of follow-up with a median follow-up time of 3.8 years (IQR: 2.4–4.6).

Median age was 33 years (IQR: 27–37), 23% had more than a primary school education, 68% were employed, and median monthly household income was 30,000 (IQR: 10,000–60,000) Ugandan Shillings (∼$12 USD). Thirty-eight percent of women were currently married or living as married. Of 133 married women, 61% reported an HIV-positive spouse, of whom 48% were on ART. Median number of prior live births was 4 (IQR: 2–6) **(** **Table 1** **)**.

**Table 1:** Baseline characteristics of female UARTO participants aged 18–49 years by pregnancy after ART initiation^1^.

	Overall (n = 351)n (%) or median(IQR)	Pregnancy after ART initiation(n = 84)n (%) or median (IQR)	No pregnancy after ART initiation(n = 230)n (%) or median (IQR)	p-value
Median follow-up (years)	3.8 (2.4–4.6)	4.1 (2.7–4.7)	4.0 (2.7–4.7)	0.891
**Socio-demographic characteristics**				
Median Age (years)	33 (27–37)	28 (24–33)	35 (30–39)	**<0.001**
Employed	240 (68%)	47 (56%)	165 (72%)	**0.020**
Education: Post- primary school	82 (23%)	16 (19%)	58 (25%)	0.623
Median monthly household income (UGX)^2^	30,000 (10,000–60,000)	30,000 (10,000–54,000)	30,000 (10,000–80,000)	0.859
Marital Status				
Married^3^	133 (38%)	45 (54%)	69 (30%)	**<0.001**
Never married	26 (7%)	10 (12%)	15 (7%)	
Widowed	89 (26%)	9 (11%)	74 (32%)	
Divorced	101 (29%)	20 (24%)	70 (31%)	
Median # livebirths	4 (2–6)	4 (2–5)	4 (3–6)	0.088
**Clinical status characteristics**				
Median time between HIV diagnosisand ART initiation (months)	14 (4–36)	13 (5–25)	15 (4–39)	0.245
Ever had AIDS Defining Illness	174 (50%)	42 (50%)	115 (50%)	0.797
BMI (kg/m^2^)				0.567
<18.5	42 (12%)	10 (12%)	24 (10%)	
[18.5–25)	238 (70%)	62 (74%)	163 (70%)	
25 or more	59 (17%)	12 (14%)	43 (18%)	
Median BMI (kg/m^2^)	21 (20–24)	22 (20–24)	21 (20–25)	0.787
Depression^4^	127 (36%)	32 (38%)	84 (37%)	0.793
Median CD4 cells/mm^3^	137 (81–207)	154 (88–230)	137 (87–199)	0.219
Virally suppressed (<400 copies/mL)	15 (4%)	2 (2%)	1 (<1%)	0.770
MOS-HIV Mental Health Summaryscore	52 (44–57)	52 (41–57)	51 (45–56)	0.790
MOS-HIV Physical Health Summaryscore	53 (44–59)	55 (46–59)	53 (43–58)	0.080
**Sexual behaviour characteristics**				
Sex in the past 3 months	155 (44%)	55 (65%)	82 (36%)	<0.001
# of sexual partners in past 3 months^5^				0.242
1	148 (95%)	54 (98%)	76 (93%)	
2+	7 (5%)	1 (2%)	6 (7%)	
Spouse HIV-positive^6^	81 (61%)	29 (64%)	39 (57%)	0.280
Spouse on ART^7^	39 (48%)	10 (34%)	22 (56%)	0.090
HIV serostatus disclosed to primary partner^8^	111 (73%)	46 (84%)	51 (65%)	0.015

1 351 women had enrolment information and are counted in the ‘Overall’ column. 314 had one or more follow-up visits (i.e., 37 women are excluded from the following two columns assessing pregnancy post ART initiation).

2 UGX is the currency symbol for Ugandan Shillings. 30,000 UGX ∼ $12.20 USD (conversion rate: 1 USD = 2,458 UGX).

3 Married or living as married, as per self-report.

4 Depression was screened using the Hopkins Symptom Check List modified for use among people living with HIV with a cut-off of ≥1.75 indicating depression.

5 Restricted to n = 155 who reported sex in the past 3 months (55 among those with pregnancy and 82 among those without pregnancy.).

6 Restricted to n = 133 who reported being currently married (45 among those with pregnancy, 69 among those without pregnancy.).

7 Restricted to n = 81 women with an HIV-positive spouse (29 among those with pregnancy, 39 among those without pregnancy.).

8 Restricted to n = 151 who reported having a spouse or regular sexual partner (55 among those with pregnancy, 79 among those without pregnancy.).

Forty-four percent of women reported sexual activity in the prior three months, of whom 95% reported only one partner and 5% reported two or more partners. Among women reporting a spouse and/or regular sexual partner, 73% had disclosed HIV status to this partner.

Median time between HIV diagnosis and ART initiation was 14 months (IQR: 4–36 months), 50% reported ever having an AIDS-defining illness, and median body mass index (BMI) was 21 kg/m^2^ (IQR: 20–24). Overall median CD4 cell count at enrolment was 137 cells/mm^3^ (IQR: 81–207), and, consistent with national guidelines to initiate therapy for pregnant women, was higher among women pregnant at enrolment (226 cells/mm^3^ (IQR: 174–397)) compared with women not pregnant at enrolment (135 cells/mm^3^ (IQR: 81–202); p<0.0001). Thirty-six percent of women screened positive for depression at baseline. Median MOS-HIV Physical Health and Mental Health Summary scores were 52 (IQR: 44–57) and 53 (IQR: 44–59), respectively.

### Pregnancy Prevalence and Outcomes at Baseline

Among 342 women with baseline data, 23 (6.7%) women reported pregnancy within 12 months prior to enrolment and 31 (9.1%) reported pregnancy at enrolment. The 31 baseline pregnancies resulted in 24 live births, two terminations/miscarriages/or stillbirths, and four women remained pregnant at censoring (1 missing outcome).

Of the 31 women pregnant at ART initiation, 63% reported HIV diagnosis more than 9 months prior to enrolment. Thus, at least 63% of the prevalent pregnancies occurred among women who knew their positive HIV status prior to conception.

### Incidence of Pregnancy after Initiation of ART

Among 314 women with at least one follow-up visit, 84 reported pregnancy over the follow-up period. Of these women, 66 reported a single incident pregnancy, 15 reported two incident pregnancies, and three women reported three incident pregnancies, totalling 105 pregnancies over 1117.6 WYs of follow-up (pregnancy incidence = 9.40 per 100 WYs; 95% CI: 7.68, 11.4). The 105 pregnancies resulted in 57 live births (54%), 22 terminations/miscarriages/or stillbirths (21%), and 12 women remained pregnant as of their most recent visit (11%) (14 missing outcomes (13%)).

**Figure 1:**
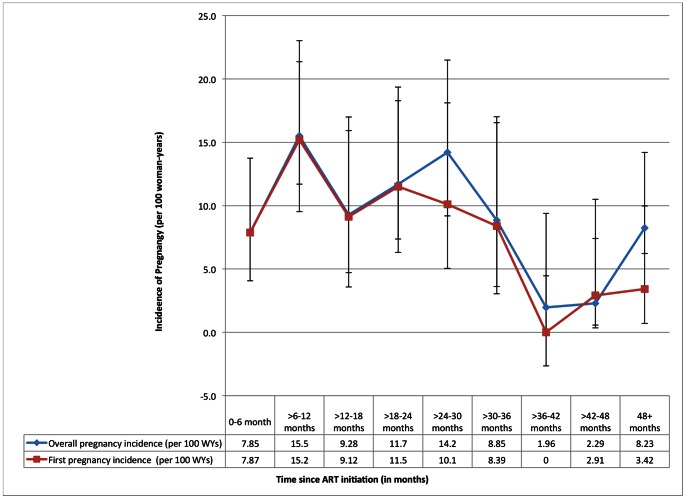
Incidence of overall and first pregnancy. Incidence of overall and first pregnancy (per 100 woman-years) by 6-month intervals post ART initiation (in months) (n = 314).

As shown in **Figure 1**, incidence of pregnancy (including first and recurrent pregnancies) was higher with recent initiation of ART. Incidence of first pregnancy peaked between 6–12 months after ART initiation (15.2 pregnancies per 100 WYS (95% CI: 9.53, 23.0)), declined and stabilized between 12–36 months, then decreased sharply 36 months after ART initiation (**Table S1**). Recurrent pregnancies peaked between 24–30 months and again at 48+ months after ART initiation. When first and recurrent pregnancy incidence are combined, overall pregnancy incidence peaked 6–12 months after ART initiation (15.5 pregnancies per 100 WYs (95% CI: 9.80, 23.2), dipped between 12–18 months, and showed a second peak from 18 through 30 months. After 30 months, we observed a steep decline in pregnancy incidence until 48+ months, when pregnancy incidence rebounded slightly. As shown in **Figure 1**, the higher overall incidence of pregnancy at 24–30 months and 48+ months was principally due to recurrent pregnancies.

### Probability of Pregnancy Over Time

**Figure 2:**
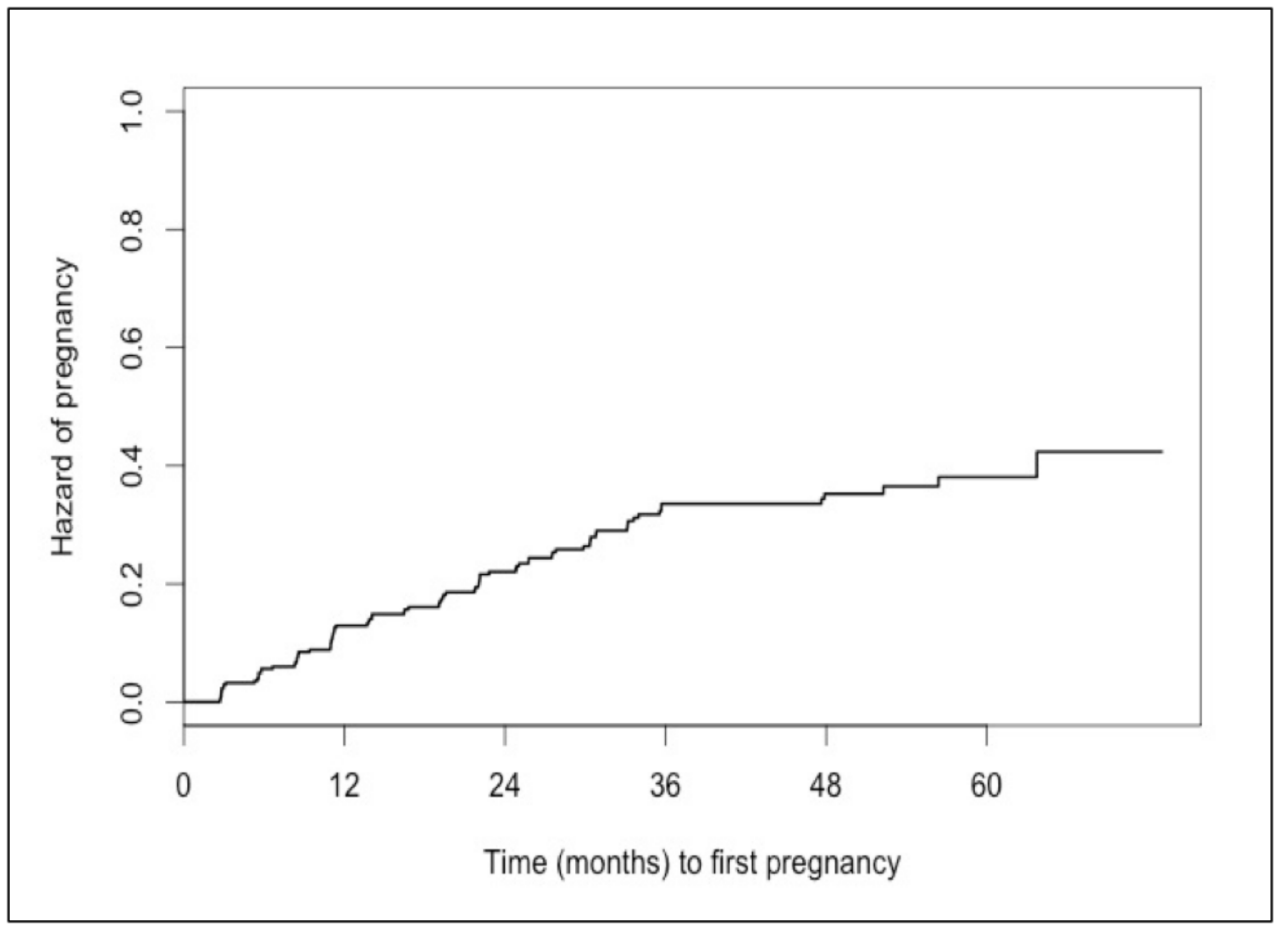
(a–d). Kaplan-Meier curves of probability of pregnancy. Kaplan-Meier curves of probability of pregnancy over time (n = 314). Figure 2a. Overall. Figure 2b. Stratified by age. Figure 2c. Stratified by marital status. Figure 2d. Stratified by disclosure. Figure 2a. Probability of pregnancy over time among HIV-positive women initiating ART. Figure 2b. Probability of pregnancy over time among HIV-positive women initiating ART, stratified by age (<35 years *vs.* > = 35 years). Figure 2c. Probability of pregnancy over time among HIV-positive women initiating ART, stratified by marital status (Currently married *vs.* not currently married). Figure 2d. Probability of pregnancy over time among HIV-positive women initiating ART, stratified by disclosure of HIV status to primary partner (Non-disclosure *vs.* Disclosure *vs*. No primary partner (N/A)).

By one, two, and three years post-ART initiation the overall probability of pregnancy was 12%, 20%, and 28%, respectively (**Figure 2** **.a**).

When stratifying by key baseline characteristics, for younger women (less than 35 years of age, the peak childbearing years for Ugandan women [43] (**Figure 2** **.b**), the probability of pregnancy after ART initiation was 18%, 29%, and 42%, by years one, two, and three respectively. Similarly, among women who were married at baseline (**Figure 2** **.c**), the cumulative probability of pregnancy by year three was 43%. Further, among women with a regular sexual partner (spouse or otherwise) to whom they disclosed their HIV status**,** the cumulative probability of pregnancy by year three was 52%. As further shown in **Figure 2** **.d**, disclosure showed a delayed effect on probability of pregnancy as it was only associated with a higher incidence among those pregnancies occurring 12 months after ART initiation.

### Baseline and Time-updated Predictors of Pregnancy Post ART-initiation

In the unadjusted analysis (**Table 2** **)**, factors associated with an increased risk of pregnancy after ART initiation included younger age (time-updated) (hazard ratio (HR): 0.90 per 1 year increase in age; 95% CI: 0.87–0.93), being married (HR: 1.93; 95% CI: 1.31–2.83), having disclosed HIV status to a primary sexual partner (time-updated) (HR: 1.90; 95% CI: 1.02–3.56), higher CD4 cell count (time-updated) (HR: 1.21 per 100 cells/uL increase; 95% CI: 1.03–1.40), and HIV diagnosis within 12–30 months of initiating ART (versus >30 months) (HR: 1.97; 95% CI: 1.14–3.41). Employed women had a lower risk of pregnancy after ART initiation (HR: 0.56; 95% CI: 0.38–0.83).

**Table 2:** Cox proportional hazard regression analysis of baseline and time-updated factors associated with pregnancy after ART-initiation (n = 314).

Variable	Unadjusted Hazard Ratio (HR) (95% CI)	Adjusted Hazard Ratio (AHR) (95% CI)
**Socio-demographic characteristics**		
Age (time-updated)	0.90 (0.87–0.93)	**0.89 (0.86–0.92)**
Employed: Yes *vs*. No	0.56 (0.38–0.83)	**–**
Education: Post-primary school *vs.* less education	0.81 (0.54–1.20)	**–**
Median monthly household income (per 10,000 UGX increase)	1.01 (0.99–1.02)	**–**
Marital Status: Married *vs.* Not married	1.93 (1.31–2.83)	‡
Number of prior Live Births:		**–**
4+ *vs*. <4	0.88 (0.60–1.30)	
2+ *vs*. 0–1 (as an alternate cut-off)	0.61 (0.37–1.01)	
**Clinical status characteristics**		
Months since HIV diagnosis		
<12 vs. more than 30	1.34 (0.80–2.23)	**–**
12–30 vs. more than 30	1.97 (1.14–3.41)	
AIDS-defining Illness (ADI) at baseline	1.24 (0.82–1.88)	**–**
BMI at baseline (kg/m^2^)	0.99 (0.94–1.05)	**–**
Depression (time-updated)	1.14 (0.77–1.70)	**–**
CD4 cell count (per 100 cells/uL) (time-updated)	1.21 (1.03–1.40)	**–**
Viral suppression (time-updated)	1.70 (0.91–3.18)	**–**
MOS-HIV Mental Health Summary score (time-updated)	0.99 (0.97–1.01)	**–**
MOS-HIV Physical Health Summary score (time-updated)	1.02 (1.00–1.04)	**–**
**Sexual behaviour**		
Spouse HIV positive at baseline		
Yes vs. No	0.95 (0.44–2.03)	**–**
N/A vs. No	0.40 (0.18–0.86)	
DK vs. No	0.74 (0.33–1.65)	
Spouse on ART at baseline	0.71 (0.37–1.40)	**–**
HIV serostatus disclosed to primary partner (time-updated)‡		
Yes vs. No	1.90 (1.02–3.56)	**2.45 (1.29–4.63)**
N/A vs. No	0.65 (0.34–1.24)	0.78 (0.41–1.50)

Notes:

‡ ‘Disclosure to primary partner’ combines information between sex with regular partner (including spouse) and disclosure. The N/A category implies that the individual does not have a regular sexual partner. Given collinearity between ‘Disclosure to primary partner’ and ‘marital status’, only ‘Disclosure to primary partner’ was included in the final adjusted model.

In the adjusted model **(** **Table 2** **)**, younger age and having disclosed HIV status to primary sexual partner (both time-updated) remained independently associated with pregnancy risk (adjusted hazard ratio (AHR) = 0.89 per year increase in age; 95% CI: 0.86–0.92 and AHR = 2.45; 95% CI: 1.29–4.63 among those who disclosed compared with those who did not disclose).

As described in the methods, ‘Disclosure to a primary partner’ combines information regarding currently having/not having a primary sexual partner (including spouse and/or regular partners) and disclosure to that partner. The ‘N/A’ category denotes that the woman does not currently have a regular sexual partner. In the adjusted model, there was no statistically significant difference in pregnancy risk between those without a primary sexual partner and those with a primary sexual partner to whom they had not disclosed HIV status, suggesting that the explanatory power of this variable stems from the presence or absence of disclosure, beyond the presence or absence of a primary sexual partner.

## Discussion

In this study, we describe the incidence of pregnancy among HIV-infected women initiating ART. Baseline pregnancy prevalence was 9% and pregnancy incidence was 9.40 per 100 WYs during a median of 3.8 years of follow-up after initiation of ART. By one, two, and three years after ART initiation, the overall cumulative probability of pregnancy in this cohort was 12%, 20%, and 28%, respectively.

Among women reporting pregnancy at baseline, over one-third were diagnosed with HIV during the pregnancy. The remaining two-thirds became pregnant after knowing their HIV status but before ART initiation. For these women, conception and pregnancy were likely associated with increased risks of poor health outcomes and of HIV transmission to sexual partners.

The observed pregnancy incidence of 9.40 pregnancies per 100 WYs is within the range of comparable regional studies of reproductive-aged women initiating ART [10], [42], [64], [65], [66]. This incidence is lower than that reported in studies employing inclusion criteria that affect probability of pregnancy, including younger age group [67] and non-use of injectable contraception [41].

We cannot directly assess whether pregnancy incidence among this cohort differs from HIV-infected women not on ART. A retrospective study of pregnancy incidence among women receiving HIV treatment and care at the referral clinic for this cohort reported a similar pregnancy incidence (8.6 pregnancies per 100 WYs) with no difference by ART use [68]. Other regional studies enrolling HIV-positive ART-naïve women have reported similar [53] or higher [69], [70], [71] pregnancy incidence. A large multi-country study found a lower incidence of subsequent pregnancy among women initiating ART during pregnancy with substantial variability in rates by individual country setting [42]. The recent results of the DART trial, which enrolled women initiating ART in Uganda and Zimbabwe, similarly reported a lower incidence of pregnancy (4.4 per 100 woman years [95% CI 4.0–4.9] [66].

The pregnancy incidence observed among UARTO participants is lower than for the general Ugandan population. The age-specific fertility rate of women aged 30–34 years in Uganda is 24.8 births per 100 women [72] which, while not directly comparable, is well above a pregnancy rate of 9.40 pregnancies per 100 WYs found in this study. This is consistent with data that suggest HIV-infected women have lower fertility than HIV-uninfected women [73], [74], [75].

The pregnancy incidence observed in this and other studies, coupled with estimates from the same site reporting that 85% of HIV-positive women do not intend to become pregnant [12], [76] but have low rates of contraceptive use [77] and regional estimates that most pregnancies among women with HIV are reported as unplanned and/or unwanted [10], [78] reinforces the need for improved, comprehensive reproductive counselling that promotes contraception to avoid unwanted pregnancies and safer conception for women who want pregnancy.

We observed that incidence of pregnancy varied with time since ART initiation, with highest incidence in periods proximal to ART initiation (with a peak in pregnancy incidence between 6–12 months) and lower incidence in periods distal to ART initiation. Four years after ART initiation, we observed a resurgence in pregnancy incidence, largely accounted for by recurrent pregnancies. Other regional studies have reported an independent effect of ART on increasing pregnancy incidence over time compared with ART-naïve women [10], [41], [42]. This is consistent with studies from North America and Europe reporting an increase in pregnancy and birth rates among HIV-positive women after widespread availability of ART [51], [54], [79], [80]. Whether increased pregnancy incidence after ART is a result of biological (e.g., improved fecundity) or behavioural change (e.g. improved sexual drive with restored health, increased fertility intentions) is not well understood but is likely due to a combination of factors [10], [42], [74], [81], [82]. These data underscore the need to incorporate comprehensive reproductive counselling for women upon HIV diagnosis and prior to ART initiation, rather than waiting and expecting women to initiate discussions with healthcare providers once they intend to become pregnant [67], [83].

Independent, time-varying predictors of incident pregnancy in this cohort include younger age and disclosure of HIV status to a primary sexual partner. Younger age has been associated with higher fertility desire [7], [9], [12], [14], [81], [84], lower contraceptive use [82], higher fecundity [85], strong societal and partner pressures towards early and frequent childbearing [81], and higher incident pregnancy in several studies of both HIV-positive women [10], [41], [42], [53] [66] and women in general [86].

As shown in Figure 2.b, among women under 35 years of age, the probability of pregnancy within three years of ART initiation was 42%, compared with 11% probability among women older than 35. While all women of reproductive age are at risk for pregnancy events and should receive routine counselling to discuss reproductive goals and services to prevent unintended pregnancies and reduce periconception-related HIV transmission risks, these data suggests that younger women are a critical target population.

Interviews with pregnant HIV-positive women in Kampala explored the complex role that HIV serostatus disclosure plays in pregnancy decision-making [87]. Disclosure is a precondition for encouraging a partner to engage in HIV risk reduction activities for the purposes of conception or otherwise and has been positively associated with partner HIV testing, increased care seeking, alleviation of anxiety, improved communication, and higher motivation to make plans for the future [88]. Women who disclose their status may encounter reduced societal and familial expectations for childbearing [81] but rising community awareness of the benefits of ART may increase pressure to conceive [89].

We observed that serostatus disclosure to the primary sexual partner was a positive and independent predictor of incident pregnancy, however, the effect was not observed until more than one year after ART initiation. Pregnancy intention was not measured in this study, however, the strong association with partner disclosure may suggest that these later pregnancies were wanted and/or planned, or discussed. Those pregnancies occurring in the first year may be highly influenced by the biological and behavioural changes observed with ART initiation and influencing fertility as described above. However, pregnancies occurring more than one-year after ART initiation might be less influenced by those changes and we may be observing a shift to more planned, intended pregnancies. The positive association between serostatus disclosure and pregnancy suggests a role for couples-based safe conception counselling in this population. More research is required to better understand this dynamic relationship.

Spouse HIV-status showed no association with pregnancy. Thirty-nine percent (39%) of women reported spouses of unknown or negative HIV-status, leaving those men at high risk for periconception or antepartum HIV-acquisition. Male partners play a large role in conception decisions and, if HIV-negative, risk HIV acquisition when seeking to conceive with an HIV-positive partner [7], [13], [90]. Comprehensive reproductive counselling programs must include men.

Limitations of this study include use of self-report for pregnancy, which likely led to an underestimate of the true incidence. Pregnancies resulting in spontaneous abortion prior to detection and pregnancies that were electively terminated may not have been fully captured. Second, we did not have data on fertility intention, pregnancy desire, or contraceptive use, which would have implications for the most appropriate intervention. A higher proportion of unplanned pregnancies would emphasize the need for integrated family planning services including a range of contraceptive options for women initiating ART, who may experience restoration of fecundity. A higher proportion of ‘planned’ or ‘desired’ pregnancies would suggest a greater role for periconception risk reduction strategies to minimize HIV transmission risks. To address these limitations we have initiated a reproductive health study within this cohort to collect these data with the goal of understanding determinants of fertility intention, behaviour, and pregnancy among HIV-affected couples to inform the design of integrated bio-behavioural interventions to mitigate HIV-transmission risk among couples who intend to have children.

### Conclusion

This study measured pregnancy incidence among HIV-positive women initiating ART and followed over a five-year period. Our findings that 9% were pregnant at ART initiation and that nearly one-third experience pregnancy subsequent to ART initiation highlight the need for integrated reproductive counselling and services that prevent unintended pregnancies and reduce periconception-related risks for HIV-positive women choosing to conceive.

## Supporting Information

Table S1 Incidence of overall and first pregnancy (per 100 woman-years) among HIV-positive women by 6-month intervals post ART initiation (n = 314).
(DOCX)
