# Correction: Incidence and Predictors of Pregnancy among a Cohort of HIV-Positive Women Initiating Antiretroviral Therapy in Mbarara, Uganda

**DOI:** 10.1371/annotation/17310bbb-e5bf-4901-8b6e-529577a280db

**Published:** 2013-06-04

**Authors:** Angela Kaida, Lynn T. Matthews, Steve Kanters, Jerome Kabakyenga, Conrad Muzoora, A. Rain Mocello, Jeffrey N. Martin, Peter Hunt, Jessica Haberer, Robert S. Hogg, David R. Bangsberg

In Figure 2, parts b, c, and d were omitted. The full, correct figure can be viewed here: 

**Figure:**
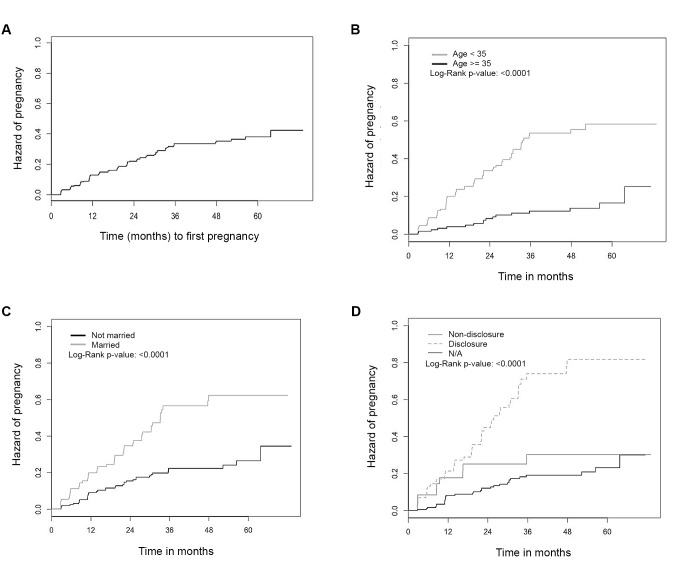


The revised legend for Figure 2 can be found below:

Figure 2. (a–d). Kaplan-Meier curves of probability of pregnancy over time among HIV-positive women initiating ART (n = 314). Figure 2a. Overall. Figure 2b. Stratified by age (35 years vs. >=35 years). Figure 2c. Stratified by marital status (Currently married vs. not currently married). Figure 2d. Stratified by disclosure of HIV status to primary partner (Non-disclosure vs. Disclosure vs. No primary partner (N/A)).

