# Supplementary material for: Incidence and Predictors of Pregnancy among a Cohort of HIV-Positive Women Initiating Antiretroviral Therapy in Mbarara, Uganda
Source: PLoS One. 2013 May 21;8(5):e63411. doi: 10.1371/journal.pone.0063411 (PMC3660357; doi:10.1371/journal.pone.0063411)
Supplement: Table S1 — Incidence of overall and first pregnancy (per 100 woman-years) among HIV-positive women by 6-month intervals post ART initiation (n = 314). (DOCX) [file pone.0063411.s001.docx]

**Table S1: Incidence of overall and first pregnancy (per 100 woman-years) among HIV-positive women by 6-month intervals post ART initiation (n=314)**

| **Time since initiating ART** | **# of women observed** | **# of pregnancies observed** | **Woman-years (WYs) of follow-up** | **Overall pregnancy incidence per 100 WYs (95% CI)** | **# of FIRST pregnancies observed** | **Woman-years of follow-up for first pregnancy** | **First Pregnancy incidence per 100 WYs (95% CI)** |
| --- | --- | --- | --- | --- | --- | --- | --- |
| 0-6 months | 314 | 12 | 152.93 | 7.85 (4.05, 13.7) | 12 | 152.49 | 7.87 (4.07, 13.8) |
| >6-12 months | 298 | 23 | 148.72 | 15.5 (9.80, 23.2) | 22 | 144.66 | 15.2 (9.53, 23.0) |
| >12-18 months | 293 | 13 | 140.06 | 9.28 (4.94, 15.9) | 12 | 131.61 | 9.12 (4.71, 15.9) |
| >18-24 months | 278 | 16 | 136.72 | 11.7 (6.69, 19.0) | 14 | 121.32 | 11.5 (6.31, 19.4) |
| >24-30 months | 268 | 18 | 127.16 | 14.2 (8.39, 22.4) | 11 | 108.68 | 10.1 (5.05, 18.1) |
| >30-36 months | 242 | 10 | 113.03 | 8.85 (4.24, 16.3) | 8 | 95.30 | 8.39 (3.62, 16.5) |
| >36-42 months | 209 | 2 | 102.26 | 1.96 (0.24, 7.07) | 0 | 82.67 | 0.00 (0.00, 4.46) |
| >42-48 months | 181 | 2 | 85.75 | 2.33 (0.28, 8.43) | 2 | 67.53 | 2.96 (0.36, 10.7) |
| 48+ months | 140 | 9 | 110.98 | 8.11 (3.71, 15.4) | 3 | 89.06 | 3.37 (0.69, 9.84) |
| **Overall** | **314** | **105** | **1117.62** | **9.40 (7.68, 11.4)** | **84** | **993.33** | **8.46 (6.75, 10.5)** |
